# The Janus face of tissue-resident memory T cells: dual programming in tumor immune surveillance and autoimmune pathology

**DOI:** 10.3389/fimmu.2026.1806804

**Published:** 2026-04-21

**Authors:** Yalin Wu, Sha Tian

**Affiliations:** 1 College of Pharmacy, Hunan University of Chinese Medicine, Changsha, Hunan, China; 2 College of Integrated Traditional Chinese and Western Medicine, Hunan University of Chinese Medicine, Changsha, Hunan, China

**Keywords:** autoimmune diseases, cellular programming, immune microenvironment, immunotherapy, tissue-resident memory T cells, tumor immune surveillance

## Abstract

Tissue-resident memory T cells (Trm) are a subset of memory T cells that establish residency in peripheral tissues and do not re-enter circulation under homeostasis, playing a central role in local immune responses. Recent studies have revealed that Trm cells play a dual role in immune protection and immunopathology: in the tumor microenvironment they can directly kill tumor cells and enhance the efficacy of immunotherapies, serving as key mediators of antitumor immunity; conversely, in autoimmune diseases they may persist long-term and drive chronic inflammation and tissue damage. This functional duality is closely linked to the microenvironmental signals, transcriptional programs, and metabolic states that shape Trm cells. This review systematically examines the developmental differentiation and molecular features of Trm cells, their bidirectional regulatory mechanisms in cancer and autoimmunity, and outlines the therapeutic potential and challenges of precision disease interventions targeting Trm cells.

## 1 Introduction

Memory T cells are pivotal for long-term adaptive immune protection. Among these, tissue-resident memory T cells (Trm) constitute a distinct subset that establishes residency in peripheral tissues (such as skin, mucosa, and parenchymal organs) without entering the systemic circulation (1). They anchor to tissues by expressing molecules such as CD69 and CD103, enabling rapid initiation of local immune responses upon pathogen re-invasion or tumor development, thereby constituting the “first line of defense” in immune protection (2, 3). However, this potent local residency and responsiveness acts as a double-edged sword: in tumor immune surveillance, Trm cells serve as potent weapons for eliminating cancer cells and improving prognosis (4, 5); yet in autoimmune diseases, their prolonged survival and abnormal activation constitute key factors driving chronic inflammation and recurrent tissue damage (6, 7). Understanding the programming and regulatory mechanisms underlying this dual nature of Trm cells is crucial for developing precise immunotherapy strategies.

## 2 Definition and characteristics of Trm cells

Trm cells constitute a memory T cell subset that resides long-term in peripheral tissues without participating in circulation. Their characteristic phenotype includes high expression of tissue-residency markers (e.g., CD103, CD69, CD49a) and low expression of circulation-associated receptors (e.g., CCR7, S1PR1, CD62L), presenting an overall state of tissue adaptation rapid responsiveness to local stimuli (1, 8). In both mice and humans, the phenotype of Trm cells is highly conserved; CD69 is considered their core marker, while CD103 is primarily expressed on CD8^+^ Trm cell subsets in certain tissues (such as the skin and intestine), mediating their anchoring to epithelial cells (9). CD103 (integrin αEβ7) directly mediates Trm cell anchoring within epithelial tissues by binding to E-cadherin expressed on epithelial cells. It further promotes the release of perforin and granzyme B, thereby enhancing local immune killing functions (10, 11).CD49a (integrin α1β1) significantly enhances Trm cell localization and prolonged retention within epithelial barrier tissues (e.g., skin, lungs) by recognizing collagen IV in the basement membrane, forming a stable local immune surveillance network (12, 13). Within tissues, Trm cells exhibit active patrolling behavior, rapidly identifying re-invading pathogens or abnormal cells. Upon activation, they secrete cytokines such as IFN-γ to issue an “early warning” to surrounding tissues, thereby enhancing innate and adaptive immune responses (14).Metabolically, Trm cells exhibit dependence on fatty acid oxidation (FAO) and maintain their survival and function by expressing fatty acid-binding proteins (FABP4/5) to uptake exogenous lipids (15, 16).

## 3 The origin and phenotypic characteristics of Trm cells

### 3.1 Differentiation pathways and tissue localization

There are several models of Trm cell differentiation, including the linear differentiation model, the branching differentiation model, and the self-renewing effector model. Existing research suggests Trm cells may derive from circulating effector memory T cells (Tem) or central memory T cells (Tcm), differentiating into a resident phenotype under the influence of local tissue microenvironment signals such as TGF-β and IL-15 (17).Stem cell memory T cells (Tscm), as an early memory subset with self-renewal capacity, may also participate in the formation of Trm (17).Research indicates that naive T cells encountering αV integrin-expressing dendritic cells in secondary lymphoid organs receive active TGF-β signaling, thereby undergoing epigenetic “pre-programming” that enhances their subsequent differentiation potential into epithelial Trm (particularly the CD103^+^ subpopulation) (14).

Although T-cell clones are evenly distributed throughout peripheral tissues and lymphoid organs during the effector phase, their differentiation fates differ during the memory phase (18). Some clones differentiate into tissue-resident memory T cells (Trm), while others form circulating memory T cells (Tcir).Further analysis revealed the presence of a subset of memory precursor cells within circulating effector T cells that exhibit Trm-like transcriptional profiles. These cells highly express Trm-associated genes such as Itgae (CD103), Itga1 (CD49a), Fabp5, Ccr10, and Ahr, and their abundance correlates positively with the subsequent capacity to form Trm. More importantly, Trm populations formed independently in different anatomical sites are highly similar in their clonal composition, indicating that the differentiation potential of Trm is a clonally inherent property established during the circulatory phase before the cells enter the tissue (18).

### 3.2 Molecular markers and transcriptional programs

The TGF-β signaling pathway plays a pivotal role in the development and maintenance of CD103^+^ Trm cells. A systematic review by Qiu et al. indicates that TGF-β, through integrin-mediated activation mechanisms, specifically regulates CD103 expression in distinct tissues, thereby influencing the resident capacity of CD8^+^ Trm cells.For instance, in the skin epidermis, keratinocytes activate TGF-β by expressing integrins αvβ6 and αvβ8, thereby promoting the formation and long-term maintenance of CD103^+^ Trm cells. Similarly, in the small intestinal epithelium, the αvβ6 integrin expressed by intestinal epithelial cells is essential for TGF-β activation and CD103^+^ Trm cell development (19).

In this intricate programming process, transcription factors such as Hobit and Blimp1 play a pivotal role; their synergistic action initiates the Trm differentiation program. Simultaneous deletion of both nearly completely disrupts the Trm developmental network across multiple tissues including skin, gut, and liver, while the generation of circulating memory T cells remains unaffected (20).They promote tissue anchoring of Trm cells by directly suppressing the expression of circulation-associated genes such as KLF2, S1PR1, and CCR7 (14). Concurrently, the mTOR signaling pathway plays a crucial role in Trm formation by facilitating the migration and local accumulation of effector T cells within mucosal tissues; inhibition of mTOR signaling diminishes Trm cell colonization capacity in mucosal sites (21).

Furthermore, Runx3 determines the fate of CD8+ T cells to differentiate into Trm cells in various non-lymphoid tissues (including the small intestinal epithelium, kidneys, skin, and lungs) by promoting the expression of genes associated with tissue retention (such as Itgae, which encodes CD103) while simultaneously suppressing genes associated with tissue exit (such as Klf2, S1pr1, and Ccr7) (22).

### 3.3 Microenvironmental signals influencing Trm cell differentiation

TGF-β sculpts the fate trajectory of CD103^+^ Trm cells through multidimensional programming mechanisms. It establishes the cellular foundation for Trm differentiation by inducing apoptosis in short-lived effector T cells (SLECs) while supporting the survival and accumulation of memory precursor effector cells (MPECs).At the molecular level, TGF-β directly initiates CD103 transcription via the Smad3 signaling pathway while indirectly consolidating its expression by antagonizing the suppression of CD103 by transcription factors such as T-bet, Eomes, and TCF1.Furthermore, TGF-β mechanistically “locks” T cells by downregulating KLF2, thereby inhibiting expression of the tissue exit receptor S1PR1. This prevents T cell egress from tissues, ultimately facilitating the stable transition to a long-term resident phenotype (19).

Retinoic acid (RA), a metabolite of vitamin A, is another microenvironmental signaling molecule—besides TGF-β—that determines the tissue-specific programming of Trm cells. Studies have shown that the differentiation of Trm cells in different organs exhibits varying degrees of dependence on TGF-β and RA. The generation of cutaneous Trm cells depends on TGF-β, while hepatic Trm cells primarily depend on RA; in contrast, the differentiation of small intestinal Trm cells requires the synergistic action of RA and TGF-β (23). At the molecular level, RA acts through its nuclear receptor RARα to regulate Trm cell differentiation. For example, in T-bet-deficient CD8^+^ T cells, increased sensitivity to RA allows these cells to bypass their dependence on TGF-β via RARα signaling and still differentiate into typical small intestinal Trm cells. RA not only influences the initial differentiation of Trm cells but is also critical for their long-term maintenance. In the small intestine, sustained RA signaling anchors Trm cells within the tissue by limiting their retrograde migration from the tissue to the draining lymph nodes via the S1P signaling pathway, thereby maintaining the stability of the local immune cell pool (23).

The cytokine microenvironment provides dynamic support for the long-term survival of Trm cells. IL-7 and IL-15 act in concert to maintain Trm homeostasis: when IL-15 signaling is absent, Trm cells become more dependent on IL-7, as evidenced by upregulated expression of IL-7Rα (CD127) (24, 25). In epithelial tissues such as the skin, Trm cells are often localized around hair follicles, where the microenvironment continuously supplies IL-7 and IL-15, which is critical for their long-term survival (24). The local metabolic environment of tissues also influences the fate of Trm cells; for example, activation of the mevalonate-cholesterol synthesis pathway, driven by the transcription factor SREBP2, promotes coenzyme Q production and enhances mitochondrial respiration, thereby supporting Trm cell survival (26).

The key molecular characteristics that distinguish Trm cells from circulating memory T cells (Tcm and Tem) lie in their “tissue-resident” transcriptional program and phenotype. Specifically, Trm cells reside in peripheral tissues by highly expressing tissue-anchoring molecules (such as integrins CD103 and CD49a) and CD69, which functions as both an activation marker and a regulator of tissue retention, while simultaneously significantly downregulating key receptors involved in lymphocyte recirculation (such as S1PR1, CCR7, and CD62L) (27). Compared to Tcm and Tem cells, which are primarily characterized by recirculation and lymphatic homing, Trm cells upregulate genes associated with tissue retention, local survival, and signaling regulation (such as Itgae, Itga1, Rgs1, and Nr4a1) and suppress the expression of the S1pr1 gene, which promotes cell migration. This programming, driven by local microenvironmental signals (such as TGF-β and IL-15), enables Trm cells to form the first line of immune defense in barrier tissues, performing rapid and long-lasting local immune surveillance (27).

### 3.4 Tissue-specific transcriptional and epigenetic programming

Trm cells exhibit significant transcriptional, epigenomic, and functional adaptations across distinct tissue environments. Crowl et al. systematically compared CD8^+^ Trm cells from mouse small intestinal epithelium, kidney, salivary gland, adipose tissue, and liver (28). They found that while these cells share a core “tissue residency program,” Trm cells from different tissues possess unique gene expression modules and chromatin accessibility profiles. For instance, Trm cells in the small intestine and salivary glands highly express TGF-β-induced genes and rely on sustained TGF-β signaling to maintain the CD103^+^ phenotype, a feature absent in Trm cells from fat, kidney, and liver tissues. The transcriptional repressor Hic1 was identified as a key regulator of small intestinal Trm cell differentiation; its overexpression enhances Trm formation in the small intestine and boosts anti-infective protection. Furthermore, Trm cells in different tissues exhibit marked differences in migratory behavior, metabolic dependency, and cytokine responses, suggesting their functional output is highly tissue-environment dependent (28).

The heterogeneity of Trm cells is not only evident across different tissues but also manifests as functional differentiation within distinct microenvironmental niches within the same tissue. Taking the small intestine as an example, Trm cells located at the tips of intestinal villi highly express effector molecules (such as Gzma and Gzmb) and possess strong cytotoxic potential; in contrast, Trm cells located at the base of crypts and in the lamina propria highly express molecules associated with long-term memory maintenance (such as Tcf7 and Id3) and form more complex cellular interaction networks with CD4^+^ T cells, B cells, and fibroblasts (29).

This functional heterogeneity is also clearly evident in the immune response to infection. Using a mouse model, Fung et al. revealed the functional division of labor among intestinal Trm subsets during secondary infection. CD103^+^ Trm cells remain stable during secondary infection, do not leave the intestine, and have limited reactivation capacity; in contrast, CD103^−^ Trm cells are the primary subset that proliferates *in situ* following local antigen stimulation and serve as the main responders during secondary infection. Transcriptomic analysis revealed that while CD103^−^ Trm cells maintain their tissue-resident characteristics, they retain some genetic features of circulating memory T cells (such as Klf2 and Eomes) and exhibit greater migratory potential, TCR signaling sensitivity, and cytokine production capacity (30).

Trm cells also exhibit differences in their functional subpopulation composition across different disease states within the same tissue. Both psoriatic arthritis (PsA) and rheumatoid arthritis (RA) patients have cytotoxic Trm cells and regulatory T cell-like Trm cells in their synovial fluid; however, PsA synovial fluid specifically enriches a population of CD161^+^CCR6^+^ IL-17A^+^ Trm cells (31). This cell population exhibits a pro-inflammatory cytokine profile while expressing low levels of cytotoxicity-associated genes (such as GZMB and PRF1). In contrast, the Trm subpopulation enriched in RA joints is characterized by high expression of cytotoxicity molecules (granzyme B, perforin) and Eomes. The TCR repertoire of IL-17A^+^ Trm cells in PsA shows low overlap with that of other Trm cells and CD8^+^CD103^−^ T cells, suggesting an independent developmental or expansion pathway. This finding highlights compositional differences in Trm subsets across different types of arthritis and provides an immunological basis for understanding their divergent clinical responses to IL-17 blockade therapy (31).

## 4 The role of Trm cells in tumor immune surveillance

### 4.1 Mechanisms of tumor recognition and clearance

Trm cells directly kill tumor cells by expressing cytotoxic molecules such as perforin and granzyme B (GzmB), and by secreting cytokines such as IFN-γ and TNF-α, they recruit and activate other immune cells, including dendritic cells and natural killer cells, thereby enhancing the local antitumor immune response (32).

For example, in colorectal cancer, patients with high levels of infiltrating double-positive (DP, CD69^+^CD103^+^) CD8^+^ Trm cells exhibit significantly longer disease-free survival and overall survival, playing a protective role in suppressing tumor invasion and lymph node metastasis (33). Mechanistically, DP CD8^+^ Trm cells highly express granzyme B, perforin, and FasL, directly killing tumor cells via an FAS/FasL-dependent pathway. Furthermore, DP CD8^+^ Trm cells highly express the chemokine CXCL13, which recruits B cells into the tumor microenvironment via the CXCL13/CXCR5 axis, promoting the formation of tertiary lymphoid structures and thereby synergistically enhancing the local antitumor immune response. Regarding differentiation regulation, the Notch signaling pathway transcription factor RBPJ interacts with the TGF-β signaling pathway transcription factor SMAD3; the two factors co-bind to the promoter region of the ITGAE gene (encoding CD103) and jointly regulate the differentiation of DP CD8^+^ Trm cells (33).

Trm cells play a key role in controlling cancer metastasis. Studies have shown that Trm cells can actively home to pre-metastatic niches and prevent distant tumor spread by suppressing tumor cells or intercepting the formation of micrometastases. Using a mouse model of melanoma-associated vitiligo, Molodtsov et al. identified tumor-specific CD8^+^CD69^+^CD103^+^ Trm cells in the skin-draining lymph nodes (32). Syngeneic experiments confirmed that this population of cells resides stably and long-term in the lymph nodes and does not participate in the recirculation. Functionally, they effectively suppress the engraftment and growth of melanoma cells within the lymph nodes, whereas circulating memory T cells lack this protective effect, indicating that lymph node Trm cells constitute a core immune barrier against tumor lymph node metastasis through local residency and effector functions (34). In a mouse breast cancer model, intervention with anti-CXCL16 antibodies promotes the migration of tumor-specific T cells from the primary tumor and their differentiation into CD103^+^CD69^+^ Trm cells in lung tissue, thereby significantly reducing the burden of lung metastases (35).

### 4.2 Factors promoting Trm cell-mediated antitumor immunity

CXCR6 has been identified as a key marker and functional regulator of Trm cells in various tumor tissues; its interaction with the ligand CXCL16 plays a decisive role in the recruitment, localization, and long-term maintenance of Trm cells (36, 37). Specifically, CXCR6 guides CD8+ T cells (including TRM precursors) to precisely localize around the DC3 dendritic cell subset in the tumor microenvironment, which highly expresses CXCL16 and IL-15Rα, thereby enabling effective reception of IL-15 signaling to sustain the long-term survival of Trm cells within the tissue; *in vivo* depletion of CD11c-positive cells leads to the rapid disaggregation and disappearance of Trm cells, confirming the critical role of this cell-cell interaction axis in maintaining TRM cells (38).

In tumor-draining lymph nodes (TDLNs), TGF-β promotes the differentiation of stem cell-like CD8+ T cells into Trm cells in a tumor-antigen-dependent manner (39). Recent studies have revealed that TGF-β induces the expression of the G protein-coupled receptor GPR25, which promotes the differentiation and expansion of Trm cells in tumor tissues such as the lungs and liver by maintaining a TCF1-dependent stem cell-like Trm program (40). Interleukin-15 (IL-15) acts synergistically with TGF-β to maintain the long-term survival of Trm in non-lymphoid tissues by regulating the expression of T-box transcription factors (such as Eomes and T-bet) (41). Dendritic cells (DCs) play a key supportive role in maintaining the Trm cell population by trans-presenting IL-15 and providing CXCL16 signaling to Trm (42).

The expression of PD-1 on Trm cells serves a dual function. On the one hand, PD-1 acts as an exhaustion marker; its binding to the ligand PD-L1 leads to functional decline in Trm cells and reduced antitumor activity (43). On the other hand, under certain conditions, PD-1 signaling can suppress T-cell migration out of tissues by downregulating sphingosine-1-phosphate receptor 1 (S1PR1), thereby helping to “lock” Trm cells within the tumor microenvironment (44, 45). In viral infection models of the skin, PD-1-deficient T cells exhibit a significant competitive disadvantage in Trm cell formation; in particular, PD-1 blockade during the early post-infection period (0–9 days) severely impairs Trm cell colonization in the epidermis and dermis. Mechanistically, PD-1 drives the early differentiation and tissue-specific programming of Trm cells and suppresses alternative differentiation fates by enhancing T-cell responsiveness to TGF-β signaling (46).

### 4.3 The impact of tumor metabolic microenvironments on Trm cell function

Tumor cells do not simply sit idly by waiting to be attacked; they influence T cells by shaping the immune microenvironment, creating a dynamic and bidirectional interaction.

On the one hand, sustained antigenic stimulation and inhibitory signals (such as the binding of the PD-1 ligand PD-L1 to PD-1, which is highly expressed on TRM cells) may cause TRM cells to enter a state of functional exhaustion (47, 48). In this state, although the cells retain the phenotype of tissue-resident cells, their functional capabilities (such as the ability to produce IFN-γ and TNF-α and to release cytotoxic granules) are impaired. On the other hand, in certain tumor models, functional dysregulation of Trm cells may exert a tumor-promoting effect. For example, excessive or inappropriate Trm cell activation may, through the release of high levels of inflammatory cytokines (such as the abnormal, sustained production of IL-17 or IFN-γ), inadvertently shape a microenvironment that favors tumor progression or immune editing, and may even induce immune-related adverse events (49–51).

In addition, while performing their cytotoxic functions, Trm cells also interact with stromal cells, dendritic cells, and myeloid cells in the microenvironment through cytokines (such as IFN-γ and IL-2) and chemokines (such as CCL3 and CCL4) (52–54). This intercellular communication helps recruit more immune cells (such as dendritic cells and circulating T cells) to the tumor site, thereby amplifying the local immune response and, to some extent, counteracting the immune-suppressive network established by tumor cells (52, 53).

Reprogramming of lipid metabolism constitutes another mechanism by which tumors subvert Trm cells. tumor cells massively uptake free fatty acids by upregulating molecules such as CD36 and the fatty acid-binding protein (FABP) family, leading to lipid depletion within the tumor microenvironment (TME). Concurrently, tumor lipid metabolism generates substantial quantities of harmful lipid derivatives, including oxidized phospholipids (OxPL). When Trm cells internalize these oxidized lipids via surface CD36, it triggers intracellular lipid peroxidation and abnormal lipid droplet accumulation. This leads to mitochondrial dysfunction and metabolic disorder, ultimately manifesting as effector function exhaustion and upregulation of inhibitory receptors (e.g., PD-1, TIM-3) (55–57). This metabolic injury exacerbates T cell exhaustion.

Ionic homeostasis disruption within the tumor microenvironment also constitutes a critical factor in Trm cell dysfunction. Studies on renal cell carcinoma (RCC) reveal markedly abnormal concentrations of ions including potassium (K^+^), sodium (Na^+^), calcium (Ca²^+^), and zinc (Zn²^+^) within the tumor microenvironment, directly impairing the ion channel function and intracellular ionic gradients of tumor-infiltrating CD8^+^ Trm cells. ICP-MS (Inductively Coupled Plasma Mass Spectrometry) and confocal microscopy analyses demonstrated elevated K^+^ and Na^+^ concentrations within tumor-infiltrating CD8^+^CD103^+^ Trm cells, inducing membrane hyperpolarization. Concurrent Ca²^+^ overload triggered mitochondrial depolarization, ultimately precipitating apoptosis. Disrupted expression of ion channel proteins (e.g., Kv1.3, KCNN4, ORAC1) further exacerbated activation deficits and diminished effector functions in Trm cells (58). These findings indicate that the tumor microenvironment actively disrupts the electrophysiological homeostasis and metabolic adaptability of Trm cells via an “ionic oscillation” mechanism, thereby undermining their anti-tumor immune function.

**Figure 1:**
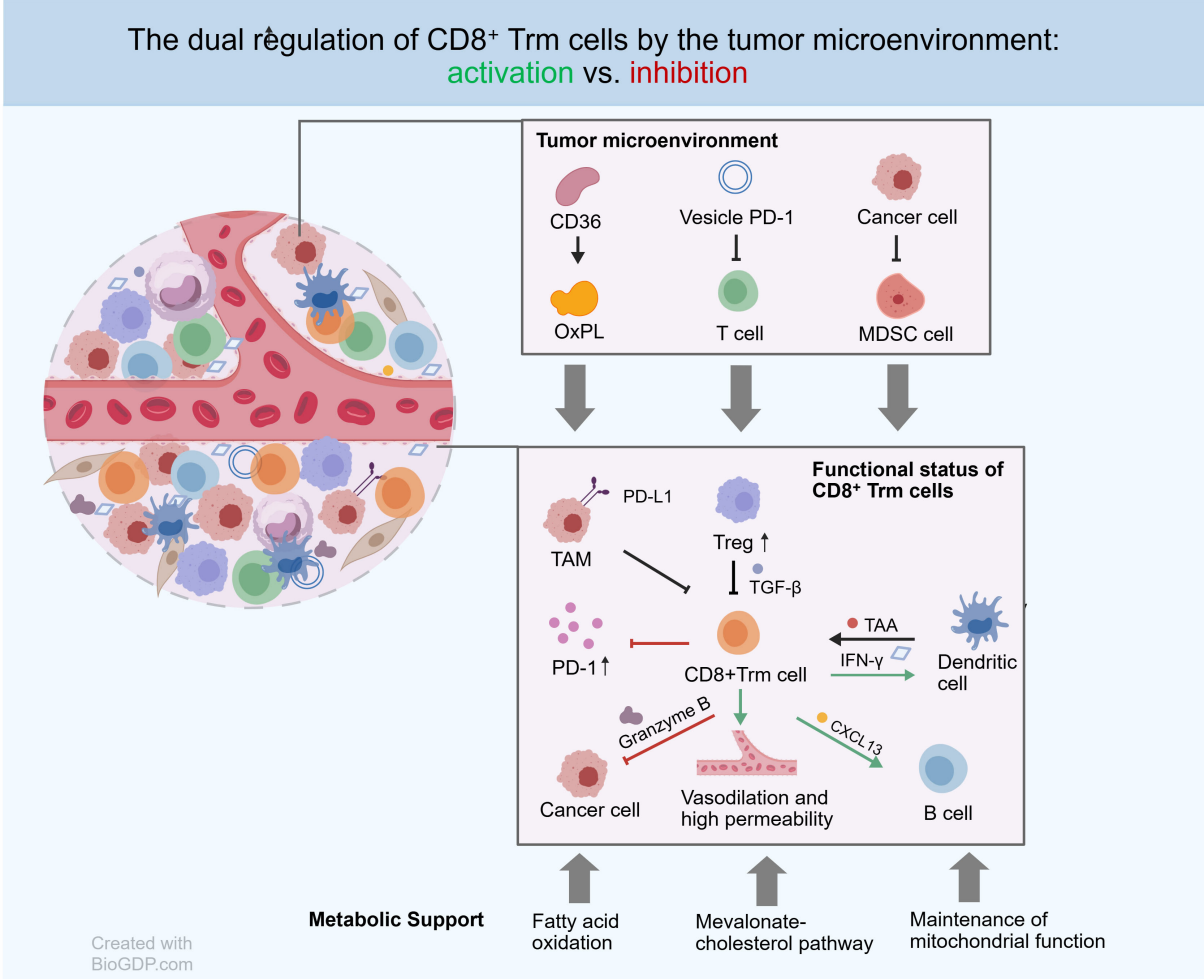
Core regulatory network of CD8^+^ Trm cell function in the tumor microenvironment. This figure illustrates the core network regulating the functional state of CD8^+^ Trm cells in the tumor microenvironment. The upper section depicts the inhibitory components of the tumor microenvironment: tumor cells upregulate CD36 to extensively uptake free fatty acids, leading to lipid depletion in the microenvironment and the production of oxidized phospholipids (OxPL); Upon uptake of OxPL, Trm cells undergo lipid peroxidation, leading to the failure of their effector functions. Tumor cells also secrete PD-1-carrying vesicles (Vesicle PD-1), which directly suppress T cell function. Myeloid-derived suppressor cells (MDSCs) further exacerbate immune suppression through metabolic competition and the secretion of inhibitory factors. The lower section depicts the functional regulatory network of Trm: Regulatory T cells (Treg) form an immunosuppressive barrier by secreting factors such as TGF-β; dendritic cells (DC) support Trm activation by presenting tumor-associated antigens (TAA) and secreting cytokines; activated Trm secrete granzyme B and IFN-γ to directly kill tumor cells and produce CXCL13, which participates in the formation of tertiary lymphoid structures. The long-term survival and functional maintenance of Trm depend on metabolic support provided by fatty acid oxidation, the mevalonate-cholesterol synthesis pathway, and mitochondrial function.

In summary, the tumor microenvironment is a dynamic ecosystem characterized by constant interplay, where the antitumor function of Trm cells represents the net outcome of their interactions with tumor cells and other immune components. This interaction forms a complex regulatory network: on one hand, TLS architecture, specific antigen presentation, and cytokines provide positive support; on the other hand, tumor-cell-driven metabolic deprivation, immune checkpoint molecules, inhibitory immune cells (e.g., Tregs), and ion homeostasis disruption collectively constitute a multi-layered inhibitory pattern, as shown in **Figure 1**.

### 4.4 Clinical significance and therapeutic potential

The clinical significance of Trm cells in various solid tumors is becoming increasingly evident, and their infiltration patterns are closely associated with patient prognosis and treatment response.

In esophageal cancer, multiple studies have confirmed the prognostic and predictive value of Trm cells. PD-1^+^CD39^+^CD8^+^ T cells infiltrating esophageal adenocarcinomas exhibit a typical CD103^+^ Trm phenotype, and their presence is significantly associated with prolonged postoperative survival in patients (59). In esophageal squamous cell carcinoma, patients with high infiltration of CD103^+^ Trm cells demonstrated greater survival benefits following treatment with nivolumab, and this marker’s predictive value was superior to that of PD-L1 expression, suggesting that Trm may be a superior biomarker for predicting the efficacy of immune checkpoint inhibitors (60).

In colorectal cancer, high infiltration of CD103^+^CD8^+^ Trm cells is not only associated with a favorable prognosis but may also serve as a potential marker for predicting the risk of liver metastasis (61). Analysis of liver metastases from colorectal cancer revealed that the CD69^+^CD103^+^ Trm subset highly expresses the tumor-reactive markers PD-1 and CD39, exhibits oligoclonal expansion, and possesses greater cytotoxicity. Its presence is independently associated with longer postoperative recurrence-free survival and overall survival, whereas the total infiltration level of CD8^+^ T cells lacks such prognostic value (62). This finding suggests that in “cold” tumors such as liver metastases, analysis of functional Trm subsets is more clinically significant than the total T-cell count. In brain metastases from gastrointestinal cancers, CD8^+^CD103^+^ Trm cells have also been shown to deeply infiltrate tumor parenchyma, and their density is positively correlated with overall survival after diagnosis, suggesting that they similarly exert antitumor effects in central nervous system metastases (63).

In melanoma, the degree of Trm infiltration is positively correlated with the accumulation of effector immune cells—such as CD8^+^ T cells, NK cells, and M1 macrophages—in the tumor microenvironment, and is significantly associated with the expression of immune checkpoint molecules and T-cell receptor diversity. Patients with high Trm infiltration exhibit significantly prolonged overall survival, and this prognostic value is independent of traditional clinical variables (64).

CD103^+^ CD8^+^ Trm cells located within mature tertiary lymphoid structures (TLS) exhibit enhanced secretion of effector molecules, such as granzyme B and IFN-γ. The combined assessment of TLS maturity and the proportion of CD103^+^ CD8^+^ Trm cells may serve as a novel biomarker for predicting prognosis in breast cancer patients (65).

## 5 The role of Trm cells in autoimmune pathology

### 5.1 Evidence linking Trm cells to autoimmune tissue damage

Trm cells exhibit abnormal expansion and sustained activation in multiple autoimmune diseases, closely correlating with chronicity and recurrence. Beyond classical studies on psoriasis and inflammatory bowel disease, recent single-cell analyses have further revealed the pivotal role of CD4^+^ Trm cells in atopic dermatitis (AD), a common chronic inflammatory skin disorder. Bai et al., analyzing single-cell transcriptomic data from adult AD lesions, identified significant enrichment of CD4^+^ Trm cells within the lesions. These cells further subdivided into functionally distinct subsets, such as JUND^+^ and RPS4Y1^+^. These CD4^+^ Trm cells highly expressed survival-related genes including FOXO1 and SBNO2, with their infiltration level positively correlated with AD severity (66).

In severe aplastic anemia (SAA), a bone marrow failure disorder, CD8^+^ Trm cells within the bone marrow microenvironment were also demonstrated to contribute to disease pathology. Through single-cell RNA sequencing analysis of peripheral blood and bone marrow mononuclear cells from SAA patients, Long et al. identified a distinct CD8^+^ Trm cell subset enriched in the bone marrow exhibiting a tissue-resident phenotype (CD69^+^CXCR6^+^) which highly express IFN-γ and FasL. These cells can induce apoptosis in hematopoietic stem and progenitor cells (HSPCs) via the FasL-Fas pathway, suggesting a key role in SAA’s immune-mediated hematopoietic destruction (67).

In rheumatic and autoimmune diseases, Trm cells also play a pathogenic role. CD8^+^CD69^+^CD103^+^ Trm cells, which are enriched in the synovium of rheumatoid arthritis (RA), activate synovial fibroblasts and promote osteoclastogenesis by secreting GM-CSF and IL-17. The disease recurrence is driven by a “lesion memory” maintained by the IL-23/IL-17 positive feedback loop (68, 69). Renal Trm cells extensively infiltrate and persistently reside in renal tissue following antigen cross-presentation by renal tubular epithelial cells. They highly express the Trm hallmarks CD69/CD103 and the inhibitory receptor PD-1, constituting a core cell population that mediates proteinuria and chronic renal tissue damage (70). In patients with primary Sjögren’s syndrome (pSS), two subpopulations of CD103^+^ and CD103^−^ Trm cells are present in the labial glands. Among these, CD103^−^ GZMK^+^ Trm cells exhibit stronger cytotoxic activity; their infiltration frequency correlates positively with disease severity (ESR, serum IgA, and focal index), making them the primary pathogenic subpopulation. CD103^+^ Trm proliferate within inflammatory foci, highly express PD-1, granzyme B, and HLA-DR, and participate in local immunopathology through tissue anchoring (71, 72).

The pathogenic role of Trm cells has been extended from peripheral tissues to the central nervous system (CNS). In brain tissue from patients with multiple sclerosis (MS) and in experimental autoimmune encephalomyelitis (EAE) mouse models, CD4^+^ Trm cells have been found to infiltrate the CNS and persist during the chronic phase (73). These CNS-resident CD4^+^ Trm cells share TCR clonotypes with circulating T cell precursors in the spleen, suggesting that pathogenic Trm cells can be replenished from peripheral precursor cells. Functional experiments demonstrate that even when peripheral CD4^+^ T cells are depleted, CD4^+^ Trm cells in the CNS can still sustain the chronic course of EAE, and their pathogenic role depends on interactions with local glial cells. The persistent presence and activation of Trm cells promote microglial activation and astrocyte proliferation, thereby maintaining a chronic inflammatory microenvironment within the CNS (73).

### 5.2 Factors driving the transition of Trm cells from protective to pathogenic phenotypes

The transition of Trm cells from a protective to a pathogenic phenotype results from functional reprogramming driven by their intrinsic plasticity under sustained abnormal microenvironmental signals. This process is primarily regulated through multiple levels, including chemokine navigation, persistent cytokine stimulation, subpopulation differentiation, and local metabolic remodeling.

At the transcriptional factor level, the hepatoleukemia factor (HLF) is a key regulator of tissue residency and the pro-inflammatory phenotype in CD4^+^ Trm cells. In CD4^+^ Trm cells, HLF suppresses genes associated with tissue exit (such as S1pr1, Klf2, Ccr7, and Tcf7) while promoting the expression of the pro-inflammatory transcription factor Bhlhe40 and effector cytokines (IL-5, IL-13, IL-17, and IFN-γ) (74). Notably, HLF expression is conserved across various human chronic inflammatory diseases—HLF^+^ CD4^+^ T cells can be detected in the pathological tissues of conditions such as chronic eosinophilic sinusitis, atopic dermatitis, inflammatory bowel disease, and primary sclerosing cholangitis, and these cells exhibit a tissue-resident phenotype and high expression of inflammatory cytokines (74).

Downregulation of the integrated stress response (ISR) pathway is one of the mechanisms underlying Trm cell dysfunction in autoimmune diseases, leading to excessive inflammatory responses. For example, in the pathological tissues of patients with anti-neutrophil cytoplasmic antibody-associated glomerulonephritis (ANCA-GN) and Crohn’s disease, the expression of ISR-related genes in CD4+ Trm cells (such as PPP1R15A, ATF3, and ATF4) is significantly downregulated, indicating reduced eIF2α phosphorylation levels and weakened inhibition of cytokine mRNA translation (75). This may lead to Trm cells continuously producing large amounts of inflammatory cytokines in the absence of adequate stimulation, thereby driving and amplifying tissue damage. In mouse models, the use of ISR pathway agonists or specific inhibition of eIF2α dephosphorylation effectively suppressed cytokine production by Trm cells and alleviated the severity of autoimmune nephropathy, providing direct evidence for targeting the ISR pathway in the treatment of autoimmune diseases (75).

Abnormal chemokine signaling guides and anchors pathogenic Trm cells. In allogeneic skin graft rejection, CD8^+^ Trm cells highly express the chemokine receptor CCR8. Blocking CCR8 or its ligands CCL1/CCL8 significantly inhibits Trm cell accumulation and activation within grafts, prolonging graft survival (76).In psoriasis recurrence, the CXCR6/CXCL16 axis has been demonstrated to be a key signal driving the retention of Tc17 subset Trm cells in the skin and the release of inflammatory factors such as IL-17 (77).

Persistent local cytokine environments disrupt Trm functional homeostasis. In patients with liver cirrhosis, IL-15 derived from peritoneal macrophages can sustainably “imprint” CD8^+^ Trm-like cells, causing them to express exhaustion markers such as PD-1 and TIM-3 while retaining high cytotoxic function (producing IFN-γ and granzyme B). This abnormally activated state may drive chronic tissue injury (7).

The pathogenic transformation of Trm cells also involves the expansion and stabilization of specific functional subsets. Single-cell studies have revealed that in clinically healed psoriatic lesions, quiescent Trm cells coexist with a population of unstable Tregs and IL-17A/F double-positive Tc17 cells, collectively forming a “cellular reservoir” for disease recurrence (78).

The interaction between Trm and Treg cells constitutes a tissue-specific immune regulatory hub that finely balances immune protection and immune tolerance in barrier tissues. Under steady-state conditions, Tregs limit the excessive activation of Trm by secreting inhibitory cytokines such as IL-10 and TGF-β. Concurrently, Trm promotes the recruitment and retention of Tregs in the tissue by secreting chemokines such as CCL17 and CCL22, forming a bidirectional regulatory loop that maintains local immune homeostasis (79, 80). However, against the backdrop of chronic inflammation or persistent tissue damage, this balance is disrupted: Treg function is impaired, and phenotypic stability declines; conversely, Trm become overactivated due to the loss of inhibitory signals from Treg, continuously secreting inflammatory factors such as IL-17, IFN-γ, and TNF-α, which further suppress Treg function, forming a positive-feedback loop of inflammatory amplification (80–82). This destabilization of the Trm-Treg regulatory axis represents a critical turning point in the transition of Trm from an immune surveillance function to a pathogenic phenotype, and serves as a key immunological basis for the persistence and recurrence of various chronic inflammatory diseases, including psoriasis, inflammatory bowel disease, and chronic obstructive pulmonary disease (83).

In summary, the pathogenic transformation of Trm cells represents a multi-signal synergistic programming process. Abnormal chemotactic signals (e.g., CCR8, CXCR6) guide their homing and retention; sustained cytokine signals (e.g., IL-15) provide abnormal activation cues; while the local microenvironment drives their differentiation into specific pathogenic subpopulations (e.g., Tc17), ultimately completing the transformation from “immune sentinels” to “inflammation drivers”.

### 5.3 The energy basis for the maintenance of Trm pathogenicity

Unlike circulating T cells, Trm cells face multiple challenges in tissues, including nutrient deprivation, hypoxia, and the accumulation of metabolic byproducts, and must reprogram their metabolic profiles to ensure a sustained energy supply (84). This metabolic adaptation not only ensures the physiological survival of Trm cells but may also serve as the energy foundation for their pathogenic transformation.

In lipid-rich tissues such as the skin, CD8^+^ Trm cells efficiently uptake exogenous fatty acids by upregulating fatty acid-binding proteins (FABP4/5) and maintain long-term survival through mitochondrial fatty acid β-oxidation (15). This metabolic program is driven by peroxisome proliferator-activated receptor γ (PPAR-γ), enabling Trm cells to persist in nutrient-limited environments. However, in autoimmune diseases, this metabolic pathway transforms into a pathogenic energy source. In patients with Crohn’s disease, intestinal CD4^+^ Trm cells exhibit enhanced fatty acid uptake and upregulated oxidative phosphorylation, acquiring an apoptosis-resistant and pro-inflammatory phenotype (85).

In the small intestine—a tissue characterized by highly active metabolite uptake—the activation and function of Trm cells are also strictly regulated by the local availability of metabolites. Konjar et al. found that intestinal epithelial lymphocytes (IELs), as typical CD8^+^ Trm cells, exist in a “semi-activated” state, exhibiting more rapid metabolic activity than circulating CD8^+^ T cells, with a close interdependence between glycolysis and oxidative phosphorylation (OXPHOS) (86). IELs are highly dependent on glucose uptake and utilization; glucose availability not only determines their glycolytic flux but also directly limits their OXPHOS activity. In the absence of exogenous glucose, the metabolic activity of IELs rapidly declines; conversely, glucose supplementation restores their oxidative metabolic capacity and enhances their effector functions, such as sustained IFN-γ production (86).

In summary, in lipid-rich tissues (such as the skin), a metabolic pattern centered on FAO is the foundation for long-term survival; however, under pathological conditions, it can evolve into a metabolic basis that drives inflammation. In contrast, in nutrient-rich tissues (such as the small intestine), the functional activity of Trm is closely coupled with glucose metabolism.

### 5.4 Molecular consolidation of Trm pathogenic memory

The differentiation, maintenance, and function of Trm cells are subject to multi-level epigenetic regulation, including chromatin accessibility and DNA methylation. These epigenetic marks not only establish the tissue-resident nature of Trm cells but may also “lock” pathogenic functions into a stable cellular state in the context of autoimmune disease.

Downregulation of the transcription factor KLF2 and its target gene S1PR1 serves as the key mechanism governing the establishment of Trm cells in peripheral tissues (87). Mechanistically, cytokines in the tissue microenvironment (such as TGF-β and IL-33) induce downregulation of KLF2 transcription via the PI3K-Akt signaling pathway, thereby closing the exit pathway for T cells and achieving early consolidation of the Trm phenotype (87).

The molecular basis of this “consolidation” process lies in the establishment of epigenetic modifications such as DNA methylation. Through whole-genome methylation profiling of CD4^+^ and CD8^+^ memory T cells (including CD69^+^ Trm cells) in human bone marrow, intestine, spleen, lung, skin, and peripheral blood, the study revealed that Trm cells exhibit highly tissue-specific epigenetic signatures (88). These epigenetic signatures primarily manifest as differentially methylated regions (DMRs) in gene regulatory regions, particularly in genes associated with tissue homing, retention, and reactivation. For example, the ITGAE gene, which encodes integrin CD103, exhibits low methylation in Trm cells in the skin and lungs, consistent with the TGF-β-dependent anchoring function of these cells in epithelial tissues; conversely, the gene encoding the chemokine receptor CCR9 shows low methylation exclusively in intestinal Trm cells, pointing to their unique potential for intestinal homing (88).

Trm’s “molecular consolidation” mechanism ensures that Trm can stably adapt to its microenvironment under physiological conditions, but it also makes it prone to transforming these adaptive processes into consolidated, self-sustaining pathogenic memories when subjected to sustained pathological signals.

## 6 Molecular and functional dichotomy: programming regulation of Trm cells

### 6.1 Shared developmental pathways underpinning divergent functional outcomes

**Figure 2:**
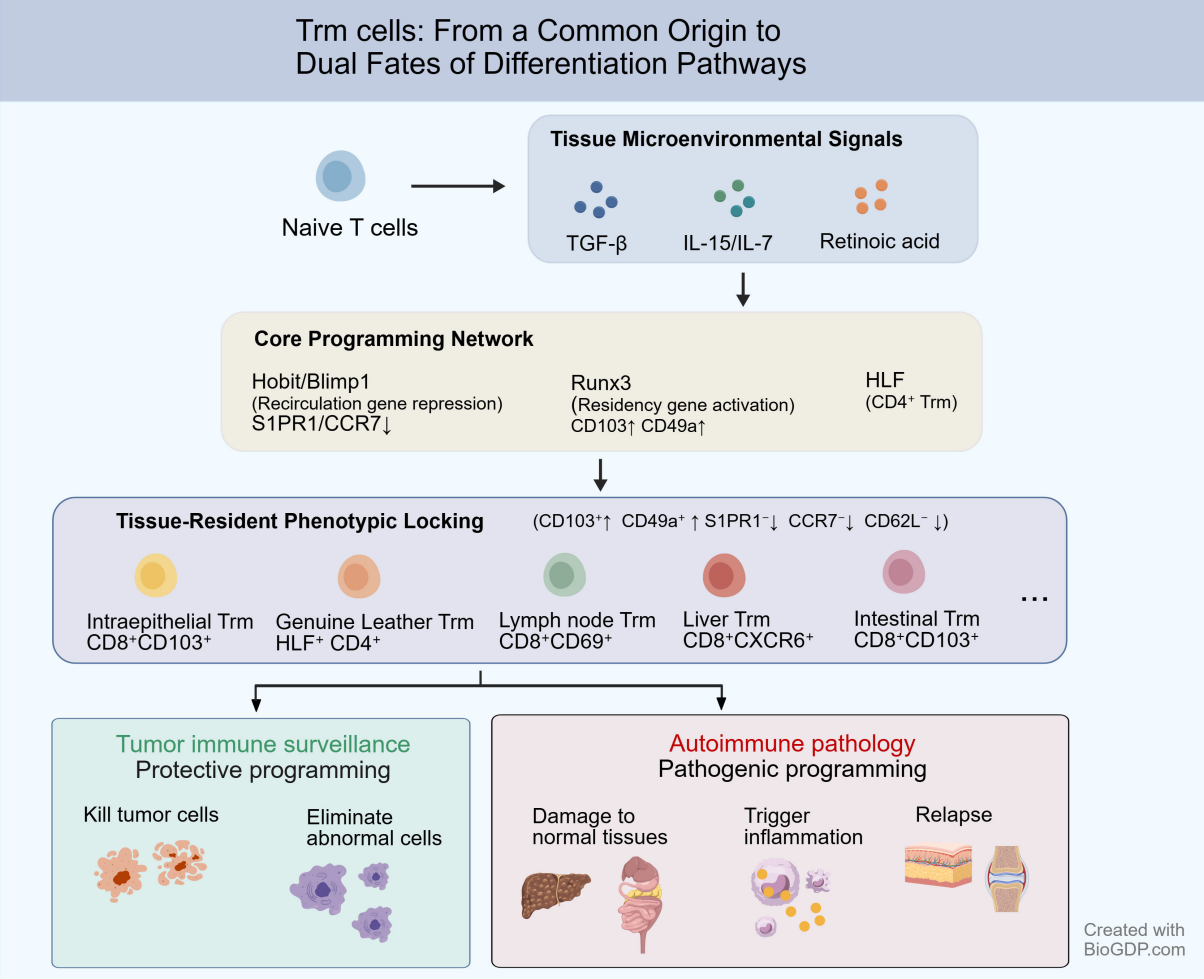
Theoretical framework of “microenvironmental programming” for Trm cell functional fate. This figure illustrates a linear differentiation model of tissue-resident memory T cells (Trm) and their functional divergence. Naïve T cells activate a core transcriptional network driven by signals from the tissue microenvironment (TGF-β, IL-15/IL-7, and retinoic acid [RA]). Hobit/Blimp1 synergistically suppress circulation-associated genes (S1PR1, CCR7) to initiate the tissue-resident program; Runx3 promotes the expression of tissue-resident genes (CD103, CD49a) while simultaneously suppressing genes associated with tissue exit; HLF (hepatocellular leukemia factor) specifically regulates the differentiation and pro-inflammatory function of CD4^+^ Trm. Upregulation of integrins CD103 and CD49a mediates cell adhesion and anchoring to epithelial cells and the basement membrane; downregulation of circulation-associated receptors such as S1PR1, CCR7, and CD62L eliminates the cells’ responsiveness to tissue-exit signals, enabling Trm to stably reside in peripheral tissues. Following phenotypic locking, Trm differentiate into tissue-specific functional subpopulations, such as intraepithelial Trm (CD8^+^CD103^+^), dermal Trm (HLF^+^ CD4^+^), lymph node Trm (CD8^+^CD69^+^), hepatic Trm (CD8^+^CXCR6^+^), and intestinal Trm (CD8^+^CD103^+^). Trm cells entering the tumor microenvironment are programmed as protective Trm, killing tumor cells and clearing abnormal cells, and are associated with a favorable patient prognosis; conversely, Trm cells entering the autoimmune microenvironment are programmed as pathogenic Trm, triggering tissue inflammation and damage, and persist as a “cell reservoir” in clinically healed lesions, leading to disease recurrence.

Trm cells exhibit diametrically opposed functional outputs in tumor immune surveillance and autoimmune pathology—eliminating abnormal cells in the former and driving tissue damage in the latter. To systematically elucidate the unified logic underpinning the dual roles of Trm cells, this paper proposes the “microenvironment programming” theoretical framework, as shown in **Figure 2**. This theory posits that the functional dichotomy of Trm cells stems from the specific reprogramming of their “tissue-resident core program” under the drive of distinct pathological microenvironmental signals. Tumor or autoimmune microenvironments provide fundamentally different sustained signals that directionally reshape the core program, ultimately giving rise to Trm subsets with opposing functions and outcomes.

The fate of Trm cells is jointly shaped by signals from their local microenvironment. Across diverse tissues and disease contexts, signals such as TGF-β and IL-15 are widely recognized as key drivers of differentiation from initial or circulating T cells toward a resident phenotype. For instance, in pulmonary infection models, local TGF-β signaling is essential for the epithelial anchoring of CD103^+^ Trm cells, while IL-15 serves as an indispensable survival signal sustaining their long-term persistence (13). Concurrently, a conserved transcriptional regulatory network governs the initiation of the ‘tissue residency program’. Transcription factors such as Hobit, Blimp1, and Runx3 are demonstrated to be widely expressed in Trm cells from diverse tissue origins. They synergistically suppress circulation-associated genes (e.g., S1pr1, Ccr7) while promoting the expression of tissue-anchoring molecules (e.g., Itgae, encoding CD103), thereby completing the phenotypic lockdown from circulating to resident cells (89).

Thus, the “Janus-faced” nature of Trm cells at developmental crossroads does not diverge onto separate paths, but rather proceeds along the same main thoroughfare. Ultimately, they are “fine-tuned” into distinct functional states—protective or pathogenic—based on the sustained signals (persistent antigens, inflammatory mediators, metabolic stress, etc.) delivered by their “tissue environmental destination”.

### 6.2 Signals determining protective and pathogenic programming

The differentiation of Trm cells is regulated by various transcription factors, such as Blimp1, Hobit, and Runx3. These factors collectively regulate the expression of genes associated with tissue residency and suppress the expression of receptors associated with tissue exit, such as S1PR1 (22, 90). It is worth noting that these signaling pathways do not exert protective effects unidirectionally; their functional output is highly dependent on the tissue microenvironment and the body’s state. Taking TGF-β as an example, under youthful or steady-state conditions, it promotes the differentiation and epithelial retention of CD103^+^ Trm cells, thereby enhancing local immune surveillance; however, in the context of aging or persistent inflammation, the same signaling pathway may lead to abnormal accumulation of Trm cells, functional decline, and the promotion of chronic inflammation and tissue fibrosis (16).

The differentiation of Trm cells is not only regulated by transcription factors but is also closely linked to the developmental trajectory of their progenitor cells. Currently, there are two main differentiation models: the systemic differentiation model posits that there are preferential progenitors in the circulation that differentiate into Trm cells; the tissue differentiation model, on the other hand, emphasizes that local microenvironmental signals drive circulating progenitors to “forced” differentiation into Trm cells (18, 63, 91, 92). Furthermore, the early-life developmental model suggests that Trm cells primarily establish tissue niches during infancy and early childhood, gradually shifting toward functional execution only in later childhood (93).

The function of protein tyrosine phosphatase non-receptor type 2 (Ptpn2) elucidates the complexity of timing-dependent signal regulation. Ptpn2 plays distinct roles at different stages of the Trm cell life cycle. During the effector differentiation phase in the early stages of acute viral infection, endogenous Ptpn2 in T cells maintains an adequate number of KLRG1^−^ precursor cells by limiting the differentiation of KLRG1^+^ terminal effector cells, thereby ensuring the normal formation of cutaneous Trm cells (94). However, during the memory phase, Ptpn2 is persistently expressed in established Trm cells and limits the intensity of their antigen-mediated responses. Ptpn2-deficient Trm cells produce higher levels of granzyme B and IFN-γ upon antigen encounter, exacerbating autoimmune dermatitis in skin inflammation models while providing stronger protection in viral infection models (94).

This “signal-environment” interaction pattern indicates that Trm cell programming depends not only on the presence of signals but also on their intensity, timing, and pathological context, thereby establishing a dynamic equilibrium between protection and pathology. These key regulatory molecules and their central roles in Trm cell fate determination are summarized in **Table 1**.

**Table 1:** Key regulatory molecules governing Trm cell fate and function.

Regulatory Category	Key molecule/ signaling pathway	Role in Trm differentiation/maintenance	Associated diseases/models	References
Metabolic regulation	Fatty acid oxidation (FAO)	Dependent on exogenous lipid uptake (Fabp4/5) for sustained long-term survival and function	tumors, Psoriasis, Autoimmune Diseases	(15, 16)
Transcriptional Regulation	Hobit/Blimp1	Inhibits expression of recirculation-associated genes (S1PR1, CCR7) and promotes expression of tissue-residency-associated genes	Multi-organ infection and tumor models	(20, 90)
	Runx3	Promotes CD103 expression, enhancing epithelial retention capacity	tumor and infection models	(22)
	HLF (Hepatic leukemia factor)	Specifically expressed in CD4^+^ Trm; suppresses tissue egress genes (S1pr1, Klf2, Ccr7, Tcf7); promotes expression of residency molecule CD69 and pro-inflammatory transcription factor Bhlhe40; drives tissue residency and pro-inflammatory function of CD4^+^ Trm	Chronic airway inflammation model, eosinophilic chronic sinusitis, inflammatory bowel disease, idiopathic pulmonary fibrosis, primary sclerosing cholangitis	(73)
Cytokine signaling	TGF-β	Induces CD103 expression, inhibits KLF2/S1PR1, promotes epithelial retention and long-term maintenance	Skin, intestinal infection and tumor models	(14, 18)
	IL-15/IL-7	Maintains Trm survival and homeostasis; IL-7 dependency increases in IL-15 deficiency	Skin, liver and peritoneal inflammation models	(7, 24, 25)
	IL-2	Synergizes with IL-15; can induce CXCR6 expression, but is weaker than IL-15 at inducing GZMK^+^CXCR6^+^ Trm precursors	Sjögren’s syndrome	(71)
Ion Channel Regulation	Kv1.3/KCNN4/ORAC1	Regulates intracellular K^+^ and Ca²^+^ gradients, influencing Trm electrophysiological homeostasis and function	Renal Cell Carcinoma tumor Microenvironment	(58)
Inhibitory Receptor Regulation	PD-1/LAG3	Highly expressed on CD69^+^CD103^+^ Trm cells infiltrating the kidney, particularly on the double-positive Trm subset	Autoimmune nephritis	(70)
Post-Translational Regulation	Integrated Stress Response (ISR) pathway (p-eIF2α/PPP1R15B)	Homeostatic p-eIF2α inhibits pro-inflammatory cytokine mRNA translation; activating dephosphorylation permits rapid translation, critical for Trm responsiveness and homeostasis	Autoimmune kidney disease (ANCA-GN), Crohn’s disease	(75)
Chemokine axis	CCR8/CCL1-CCL8	Promotes the recruitment and activation of CD8^+^ Trm cells in transplant rejection	Skin graft rejection model	(76)
	CXCR6/CXCL16	Mediates Trm retention and activation in skin, driving psoriasis recurrence	Psoriasis Relapse Model	(77)

Fabp4/5, Fatty acid-binding protein 4/5; ISR, Integrative stress response; p-eIF2α, Phosphorylated eukaryotic translation initiation factor 2α; ANCA-GN, Antineutrophil cytoplasmic antibody-associated glomerulonephritis.

### 6.3 Plasticity and stability of Trm cell phenotypes

Studies have shown that under certain conditions, Trm cells can re-enter the circulation or migrate to draining lymph nodes, demonstrating a degree of plasticity; however, their long-term persistence in tissues remains dependent on sustained local antigenic stimulation or tissue-specific signals (95, 96). For example, Trm cells derived from the skin can downregulate residency markers such as CD69 and enter the bloodstream, becoming “ex-Trm” cells, which are characterized by the expression of the skin homing receptor CLA and the retention of tissue-adaptive transcriptional features (such as GATA3) and metabolic molecules (such as FABP5) (97–99). Under steady-state conditions, a small number of ex-Trm cells persist in the circulation; during skin inflammation or viral infection, this population expands significantly, suggesting that these cells can be mobilized to participate in systemic immune responses (98, 100).

Studies on the migratory capacity of Trm cells have further deepened our understanding of their plasticity and stability. Through syngeneic co-culture experiments and *in vivo* migration tracking, Shou et al. demonstrated that CD8^+^ Trm cells in lung tissue can migrate retrograde through lymphatic vessels to draining lymph nodes, forming a population of “lymph node Trm” cells that exhibit a tissue-resident phenotype while residing within lymphoid organs (101). Transcriptomically, these cells occupy an intermediate position between lung Trm cells and splenic memory T cells, possessing both resident and re-responsive potential. Notably, despite their spatial migration, these cells retain the typical Trm phenotype of CD69^+^CD103^+^ and do not participate in systemic recirculation, demonstrating a unity of plasticity and stability. Trm cells in these draining lymph nodes can replenish the pulmonary Trm pool during reinfection, forming a “reverse reservoir” mechanism that provides dynamic replenishment for the long-term persistence of tissue Trm while maintaining local immune memory (101).

Behind this characteristic, epigenetic mechanisms serve as a “molecular memory bank”: by maintaining chromatin accessibility and regulating DNA methylation patterns and histone modification imprints, Trm cells are able to keep effector genes in a “standby” state during the resting phase without antigen stimulation—so that once they encounter an antigen challenge again, they can rapidly restart transcription and efficiently perform cytotoxic or cytokine secretion functions (102). For example, following influenza virus infection, the hypomethylated state of the IFITM3 gene promoter region in CD8^+^ Trm cells in lung tissue enables sustained expression of interferon-induced transmembrane protein 3, thereby providing long-lasting protection against subsequent viral infections (103). Similarly, in tumor-infiltrating CD8^+^ Trm cells, low methylation in the enhancer region of the key effector molecule perforin (PRF1) gene ensures potent cytotoxic activity (104).

The plasticity of Trm cells is also reflected in the dynamic evolution of their phenotype in response to the tissue microenvironment and host age. Analysis of Trm cells in human kidney tissue revealed significant differences in phenotype and spatial distribution between the two Trm subsets—CD69^+^CD103^+^ and CD69^+^CD103^−^: the CD103^+^ subset highly expresses CD49a and granzyme B, is sparsely distributed, and tends to exert cytotoxic effects; whereas the CD103^−^ subpopulation is characterized by CD28 expression and is predominantly clustered near monocytes/macrophages, suggesting its potential involvement in local immune regulatory networks (105). With advancing age, the proportion of the CD28^−^ subpopulation within CD8^+^CD69^+^CD103^+^ Trm cells in the kidney increases significantly, indicating that the phenotypic evolution of Trm cells is closely associated with immunosenescence (105).

## 7 Therapeutic implications and future directions

### 7.1 Cancer immunotherapy

Trm cells play a central role in tumor immune surveillance and are key targets for cancer immunotherapy. Current tumor treatment strategies targeting Trm cells primarily include the following categories:

Immune checkpoint inhibitors, such as PD-1/PD-L1 inhibitors, can promote the proliferation of Trm cells at the tumor site and enhance their cytotoxic function (106). This blockade can also indirectly enhance Trm cell function by modulating their interaction networks with other immune cells. The PD-1/PD-L1 signaling pathway directly contributes to the maintenance of Trm cell homeostasis by targeting the co-stimulatory receptors CD27 and ICOS; PD-L1 blockade can restore the function of PD-1-high Trm cell populations via the CD27/ICOS-NR4A1 axis, thereby influencing Trm-mediated tissue protection and pathological processes (107). PD-1 also mediates the early differentiation and tissue programming of cutaneous CD8^+^ Trm cells by promoting TGF-β signaling responses and skin colonization (46).

Regarding metabolic intervention strategies, Trm cells rely on fatty acid oxidation to survive in the tumor microenvironment; targeting lipid metabolic pathways (such as FABP4/5 and CD36) can selectively eliminate inhibitory Trm cells or enhance their antitumor function (57, 108). Furthermore, T-cell senescence can be reversed by inhibiting p38-MAPK signaling, enhancing autophagy (e.g., using spermidine or autophagy agonists), or improving mitochondrial function (e.g., NAD+ supplementation), thereby enhancing the survival and antitumor activity of Trm cells (109–112).

Epigenetic modulators (such as EZH2 inhibitors and DNMT inhibitors) can reverse T-cell exhaustion and enhance the memory function and antitumor persistence of Trm-like cells (113). Regarding vaccine and dendritic cell therapies, CD103^+^ dendritic cells or TGF-β-expressing dendritic cell subsets can effectively induce Trm differentiation (111). Cell-based adoptive therapy enhances the residency and responsiveness of Trm cells within tumors by infusing pre-programmed Trm cells or T memory stem cells (111). Engineered T-cell strategies also offer new approaches to enhancing Trm-mediated antitumor immunity. By co-expressing Flt3L and XCL1 (FX) on T cells, these strategies significantly enhance the recruitment and activation of dendritic cells (particularly cDC1) in the tumor microenvironment, promoting antigen diffusion and endogenous polyclonal T-cell responses, thereby providing favorable conditions for the local expansion and functional activation of Trm cells (114).

Interventions targeting the microbiome-immune axis represent a new direction in cancer immunotherapy. A mouse model of primary gastric cancer has demonstrated that eliminating or modulating harmful tumor-associated microbiota (such as Metanibacter) can effectively restore TGF-β expression levels in the tumor microenvironment, thereby promoting the generation and infiltration of CD8^+^ Trm cells and ultimately enhancing the antitumor immune response (115).

Regarding nano/microparticle delivery systems based on biodegradable materials such as PLGA, Li et al. developed PLGA nano/microparticles (OVA-aPD1 N.M.P.) loaded with OVA antigen and anti-PD-1 antibody, which successfully induced the expansion and activation of CD8^+^ Trm cells in the liver in a mouse model of hepatocellular carcinoma (116).

### 7.2 Prevention and control of infectious diseases

Trm cells form the first line of defense in mucosal and barrier tissues, and Trm-targeted strategies against viral, bacterial, and parasitic infections are gradually being developed.

In the field of antiviral research, particularly regarding latent infections such as herpesviruses, strategies targeting Trm have demonstrated unique potential. Through methods such as local mucosal vaccination, vaccine vectors expressing viral antigens, and the local application of chemokines (e.g., CXCL9/CXCL10), pathogen-specific Trm pools can be actively induced and expanded at infection gateways to establish a local immune barrier (117, 118). Concurrently, to address Trm functional exhaustion caused by the reactivation of latent viruses (e.g., high expression of PD-1 and LAG-3), the use of immune checkpoint inhibitors (such as anti-PD-1 and anti-LAG-3 antibodies) can restore Trm effector function and control viral recurrence (117). These strategies aim to leverage the long-term resident nature of Trm to achieve long-term immune surveillance and treatment for latent and recurrent infections.

In bacterial infections, Trm also plays a key role in mucosal immune protection. For example, against Klebsiella pneumoniae, researchers developed a bivalent mucosal vaccine based on the outer membrane protein OmpX and the LTA1 adjuvant, which successfully induced the generation of pulmonary CD4^+^ Trm cells through intrapulmonary vaccination; these cells mediate protective immunity via the IL-17R signaling pathway (119). Similarly, an intranasal vaccine targeting Pseudomonas aeruginosa can also induce the formation of pulmonary Trm and enhance neutrophil recruitment, thereby providing immune protection (120). These studies demonstrate that through the rational design of vaccines (such as selecting appropriate antigens, adjuvants, and routes of administration), tissue-specific Trm responses can be specifically induced, thereby establishing a long-lasting mucosal immune barrier.

In parasitic infections, intrahepatic CD8^+^ Trm play a critical role in malaria models by directly killing malaria-infected hepatocytes through the secretion of perforin/granzyme B. The absence of Trm leads to a significant increase in parasite burden (121). Furthermore, cutaneous CD4^+^ Trm cells enhance anti-parasitic immunity in Leishmania infections by rapidly secreting IFN-γ to activate macrophages and by delivering cytotoxic signals through direct contact with infected cells (122). Although the mRNA-LNP vaccine encoding the Leishmania antigen PEPCK, when used alone, can elicit a systemic Th1 response, it fails to effectively induce the formation of cutaneous CD4^+^ Trm cells. When administered in combination with an IL-12 mRNA-LNP, this significantly promoted the expression of skin homing molecules and Trm markers (CXCR6, CD69) on antigen-specific CD4^+^ T cells, established a widespread population of dTrm cells in the skin, and mediated delayed-type hypersensitivity reactions and protection against Leishmania (123).

### 7.3 Interventions for autoimmune diseases

Intervening in the metabolic reprogramming processes underlying the abnormal activation and persistence of Trm cells has emerged as a new strategy for mitigating autoimmune pathology.

In terms of metabolic intervention, the dependence of Trm cells on fatty acid oxidation provides a new therapeutic target for autoimmune diseases. As a CPT1a inhibitor, etomoxib can impair the energy metabolism of pathogenic CD8^+^ Trm cells by blocking fatty acid oxidation. The study utilized a polydopamine-etomoxib-macrophage membrane@microneedle (PEM@m) delivery system to achieve targeted inhibition of CD8^+^ Trm in a psoriatic mouse model, alleviating skin lesions and preventing recurrence, with efficacy superior to calcipotriol (124). Furthermore, a phase III clinical trial of the high-dose IL-23 inhibitor risenlimab demonstrated that this treatment significantly reduced the number of CD8^+^ Trm in skin lesions of psoriasis patients, with some patients maintaining complete clearance even 84 weeks after discontinuation, suggesting that targeting Trm metabolic signaling pathways can achieve long-term disease remission (125).

In terms of autophagy and energy metabolism regulation, the Centipede Detox Decoction can activate the inhibited Rheb/mTOR signaling pathway in psoriatic lesions, thereby reversing the state of excessive autophagy, cutting off the energy supply to pathogenic CD8^+^CD103^+^ Trm cells, effectively reducing their local numbers, and decreasing the secretion of key inflammatory factors such as IL-17, IL-22, and IFN-γ, ultimately improving lesion recurrence and dermal structural disruption (126).

Precision metabolic regulation based on nanomaterials has also shown promise; for example, in psoriasis models, nano-graphene oxide (NGO) can restore mitochondrial function and reduce levels of mitochondrial reactive oxygen species (mtROS), thereby inhibiting STAT3 phosphorylation and IL-17 expression. This reduces the accumulation of pathogenic CD8^+^CD69^+^CD103^+^ Trm cells in the skin and promotes the expansion of regulatory T cells, thereby improving pathological manifestations of the disease (127).

JAK-STAT pathway inhibitors (such as tofacitinib) can block cytokine signaling, including that of IL-15, thereby inhibiting Trm activation and inflammatory responses; they are currently widely used in the treatment of autoimmune diseases such as psoriasis and rheumatoid arthritis (128).

### 7.4 Challenges in selectively modulating Trm cell function

Despite demonstrating potential in tumor immunotherapy and autoimmune disease control, the clinical application of targeting Trm cells faces multiple challenges. Trm cells exhibit high tissue specificity and phenotypic heterogeneity. Trm cells from different tissues (such as skin, intestine, liver) or even from different diseases within the same tissue (such as psoriasis and melanoma) may express distinct markers and functional molecules, complicating the development of universal targeting strategies (22). Second, the phenotypic overlap between Trm and Tex cells (such as the expression of both CD69 and CD103) implies that, if imprecise markers are used, targeted therapy may inadvertently damage beneficial Trm cells or fail to target harmful Tex cells (129). Trm cells and circulating T cells share developmental precursors and signaling pathways; targeted interventions may compromise systemic immune memory, increasing infection risk or triggering autoimmunity (18). Furthermore, existing detection methods struggle to dynamically track Trm cell numbers and function *in vivo*, particularly lacking non-invasive imaging or biomarkers in humans. Moreover, while the metabolic plasticity of Trm cells (e.g., dependence on fatty acids and glucose) presents potential intervention targets, metabolic modulators often lack tissue specificity and may induce systemic side effects (130). Furthermore, some Trm cells simultaneously express both activating and inhibitory receptors (e.g., PD-1, TIM-3,LAG-3), with functional states dynamically shifting within the microenvironment. Single-target interventions may prove insufficient to reverse their pathological state (67). Consequently, future efforts must develop tissue-specific delivery systems, employ multi-omics technologies to decipher Trm subpopulation heterogeneity, and establish dynamic monitoring frameworks to achieve precise and safe functional regulation.

### 7.5 Emerging research frontiers and potential intervention approaches

With the continued advancement of single-cell multi-omics, spatial transcriptomics, and *in vivo* imaging technologies, research on Trm cells is entering a new phase characterized by higher resolution and dynamic visualization. Future research directions include:

At the cellular atlas level, future research will focus on constructing dynamic spatiotemporal atlases of Trm cells. By leveraging technologies such as scRNA-seq and CITE-seq, researchers will systematically analyze the evolution of their transcriptomic profiles and proteomic phenotypes across different disease stages, thereby enabling the precise identification of disease-specific functional subpopulations (66, 67). At the regulatory mechanism level, research will explore how metabolites (such as lactate and acetyl-CoA) determine the fate of Trm cells by influencing pathways such as histone modification and DNA methylation, and develop corresponding small-molecule intervention targets based on these findings.

In terms of technological translation, the development of *in situ* editing and targeted delivery systems is gradually coming into focus. Using CRISPR-Cas9 or mRNA-LNP technology, it may be possible in the future to achieve genetic modification or drug intervention of local Trm cells within tissues, thereby reshaping the local immune microenvironment (114). This approach could also provide new pathways for optimizing engineered T-cell therapies, such as designing CAR-T cells that co-express specific chemokines (e.g., XCL1) or cytokine receptors (e.g., IL-15Rα) to enhance their ability to colonize and maintain function at tumor or infection sites.

Furthermore, the integration of artificial intelligence and computational modeling is expected to introduce new research paradigms in this field. By simulating and predicting the behavioral dynamics of Trm cells in different microenvironments and their response patterns to therapeutic interventions, these approaches may eventually provide a decision-making basis for designing personalized immunotherapy regimens.

These emerging approaches are expected to overcome the current bottlenecks in Trm cell-targeted therapy and provide more precise immunotherapeutic strategies for cancer, autoimmune diseases, and transplant rejection. **Table 2** summarizes the current main therapeutic strategies, their mechanisms of action, and corresponding clinical applications.

**Table 2:** Trm-targeting intervention strategies and their mechanisms.

Treatment Category	Intervention strategy	Mechanism of action	Disease application scenario	Research stage	References
Cancer Immunotherapy	Ion Channel Modulators	Restores K^+^, Ca²^+^ and other ion homeostasis, improving Trm mitochondrial function and survival	Renal cell carcinoma	Preclinical studies	(58)
	PD-L1 blocking antibodies	Downregulate Fabp4/5 expression in tumor cells; restore Trm lipid uptake and survival; enhance anti-tumor immunity	Gastric cancer	Preclinical/Clinical	(108)
	Epigenetic regulators (EZH2 inhibitors)	Reverse the epigenetic memory of T cell exhaustion; enhance the durable anti-tumor function of Trm-like cells	Tumor immunotherapy	Preclinical/Early clinical	(113)
	Engineered T cells (FX-CAR)	Express Flt3L and XCL1; enhance DC recruitment and antigen spreading; promote Trm activation	Solid tumors (e.g., melanoma, gastric cancer)	Preclinical	(114)
	Microbiome-immune axis intervention	Modulate gastric microbiota (e.g., Methylobacterium); restore TGF-β signaling and CD8^+^ Trm infiltration	Gastric cancer	Preclinical	(115)
	OVA-aPD1 N.M.P (PLGA nanoparticle/microparticle vaccine)	Co-deliver OVA antigen and aPD1; enhance DC antigen presentation; activate non-tumor-antigen-specific CD8^+^ Trm; restore anti-tumor immunity via intercellular communication	HBV-related hepatocellular carcinoma	Preclinical	(116)
Prevention and Control of Infectious Diseases	CXCR3 signaling modulation	Regulates the CXCL9/CXCL10-CXCR3 axis, influencing CD8^+^ T cell localization in infected tissues (e.g., small intestine). CXCR3 deficiency causes Trm to accumulate in the intraepithelial compartment at the villus tips, enhancing their differentiated phenotype (Gzma^+^, Itgae^+^).	Viral infection model (LCMV); mucosal immunity	Preclinical	(118)
	OmpX + LTA1 intrapulmonary mucosal vaccine	Induces CD4^+^ Trm cells (TH1/TH17) in lung tissue; activates fibroblasts via IL-17R signaling; promotes neutrophil recruitment and local immune barrier formation	Klebsiella pneumoniae infection (including hypervirulent and drug-resistant strains)	Preclinical	(119)
	Intranasal rePcrV + curdlan vaccine	Induces CD4^+^ Trm cells (primarily secreting IL-17A) in lung tissue; promotes neutrophil recruitment and local immune barrier formation via IL-17A signaling; protection is independent of IL-7 signaling	Pseudomonas aeruginosa pneumonia (including drug-resistant strains)	Preclinical	(120)
	Anti-ARTC2.2 nanobody (s+16a)	Blocks ARTC2.2-mediated, P2X7-dependent cell death before hepatic Trm isolation; improves Trm survival for functional analysis and adoptive transfer studies	Malaria	Preclinical	(121)
	Skin CD4^+^ Trm induction (natural infection)	Leishmania infection induces IFN-γ^+^ CD4^+^ Trm cells in the skin, which persist long-term without continuous antigen stimulation; upon reinfection, they rapidly recruit circulating T cells via a CXCR3-dependent pathway, enhancing local immune control	Cutaneous leishmaniasis	Preclinical	(122)
	IL-12 mRNA-LNP + PEPCK mRNA-LNP vaccine	IL-12 promotes Th1 differentiation; upregulates skin-homing molecules (PESL); induces CD4^+^ dTrm cell development; enhances local skin immunity	Cutaneous leishmaniasis	Preclinical	(123)
Interventions for Autoimmune Diseases	Chemokine receptor blockade (anti-CXCR6)	Inhibits the CXCL16-CXCR6 axis; reduces Tc17 Trm retention and activation in the skin	Psoriasis recurrence	Preclinical	(77)
	PEM@m Microneedle Patch (macrophage membrane + Etomoxir)	Macrophage membrane adsorbs various inflammatory cytokines (IL-6, CXCL1, etc.) and inhibits NET formation; Etomoxir inhibits CPT1a, blocking fatty acid oxidation and eliminating CD8^+^ Trm; downregulates pentose phosphate pathway (PPP) activity, reducing CD8^+^ Trm survival; PDA scavenges ROS, enhancing local anti-inflammatory effects.	Psoriasis	Preclinical	(124)
	Risankizumab	High-dose IL-23 inhibitor; significantly reduces the number of CD8^+^ TRM17 cells in lesions; downregulates IL-17A/F/IL-22 expression; disrupts the IL-7/IL-15/TL1A signaling axis between keratinocytes and Trm cells; enables long-term remission after drug withdrawal.	Psoriasis	Phase II Clinical	(125)
	Traditional Chinese Medicine Compound (Wugong Beiduyin)	Regulates the Rheb/mTOR autophagy pathway; inhibits energy supply to Trm cells; reduces inflammatory factor release.	Psoriasis recurrence	Preclinical	(126)
	JAK-STAT pathway inhibitors	Inhibit cytokine signaling (e.g., IL-15); regulate Trm activation and inflammatory responses	Autoimmune diseases, Tumors	Widely used in clinical practice	(128)

PLGA, polylactic-co-glycolic acid; OVA, ovalbumin; aPD1, anti-PD-1 antibody; CPT1a, carnitine palmitoyltransferase 1a; JAK-STAT, Janus kinase-signal transduction and transcription activator; LNP, lipid nanoparticle; Th1, type 1 helper T cells.

## 8 Conclusions

### 8.1 Dual role of Trm cells

The functional output of Trm cells is highly dependent on signals from their microenvironment, transcriptional programs, and metabolic state. Within the tumor microenvironment, Trm cells directly kill tumor cells and recruit other immune cells by expressing cytotoxic molecules (such as perforin and granzyme B) and secreting cytokines such as IFN-γ and TNF-α, thereby forming the first line of local immune defense. Clinical studies have shown that the infiltration of CD8^+^CD103^+^ Trm cells within tumors is positively correlated with improved patient prognosis and response to immunotherapy. However, in autoimmune diseases such as psoriasis, atopic dermatitis, and severe aplastic anemia, Trm cells undergo abnormal expansion, persist for extended periods, and remain continuously activated. By secreting inflammatory factors such as IL-17 and IFN-γ, they drive chronic tissue damage and disease recurrence. This dual role of “protection and pathology” highlights the complexity and plasticity of Trm cells in immune homeostasis.

The core similarities and differences in Trm cell function, molecular mechanisms, and intervention strategies within tumor immunity and autoimmunity are summarized in **Table 3**.

**Table 3:** Dual roles, regulatory mechanisms, and therapeutic strategies of Trm cells.

Dimension	Tumor immunity (protective role)	Autoimmunity (pathogenic role)
Primary Function	Directly killing tumor cells, enhancing immunotherapy response, forming local immune barriers	Drives chronic inflammation, tissue damage, and disease recurrence
Key Molecular Markers	CD103^+^, CD69^+^, CXCL13^+^, Granzyme B^+^, IFN-γ^+^	CD69^+^, CD103^+^, IL-17^+^, CXCR6^+^, CCR8^+^
Regulatory mechanisms	TGF-β signaling, IL-15/IL-7, Hobit/Blimp1, glycolysis/FAO metabolism, epigenetic priming	Abnormal chemokine signaling (CXCL16-CXCR6, CCL1/CCL8-CCR8), sustained IL-15 stimulation, metabolic reprogramming (Fabp4/5)
Representative Diseases/Models	Melanoma, esophageal cancer, renal cell carcinoma, breast cancer and colorectal cancer, etc.	Psoriasis, atopic dermatitis, severe aplastic anemia, lupus nephritis, skin graft rejection etc.
Clinical relevance	Positively correlated with favorable prognosis and immunotherapy response (e.g., melanoma, lung cancer, gastric cancer)	Positively correlated with disease severity and recurrence risk (e.g., psoriasis, AD, SAA, etc.)
Therapeutic strategies	Immune checkpoint inhibitors, vaccine-induced Trm, engineered T cells (FX-CAR), metabolic interventions, epigenetic modulators	Targeted therapies against chemokine receptors (e.g., CXCR6, CCR8), metabolic inhibitors (e.g., Fabp4/5), traditional Chinese medicine formulations, local nanodelivery systems, JAK-STAT inhibitors, and IL-23 inhibitors (e.g., risenlimab)
Challenges and Outlook	Tissue heterogeneity, metabolic plasticity, limited diagnostic tools, optimization of combination therapy strategies	Target specificity, safety, long-term efficacy monitoring, clinical translation pathways

### 8.2 The clinical significance of Trm cell programming

The differentiation and functional regulation of Trm cells involve multidimensional programming mechanisms, including transcription factor networks (e.g., Hobit, Blimp1, Runx3), cytokine signaling (TGF-β, IL-15, IL-7), metabolic reprogramming (fatty acid oxidation, glycolysis), and epigenetic regulation. Epigenetic regulatory mechanisms provide novel molecular insights into the dual functions of Trm cells. In tumor immunity, maintaining the epigenetic “primed” state of Trm cells enhances their sustained anti-tumor capacity; conversely, in autoimmunity, aberrant epigenetic memory may lead to pathological persistent activation of Trm cells (97).Alterations in three-dimensional genomic architecture (e.g., chromatin intradifferentiation) also influence Trm cell functional stability and pathological transformation potential (97). These programming mechanisms offer potential targets for developing disease-specific immune intervention strategies.

### 8.3 Concluding outlook on balancing immune protection and pathological damage via Trm cell regulation

Future therapeutic strategies targeting Trm cells should adhere to the principles of “precise regulation, localized intervention, and dynamic monitoring”. Approaches such as epigenetically-based reprogramming strategies (e.g., EZH2 inhibitors, DNMT inhibitors) (106), metabolic interventions (e.g., targeting fatty acid oxidation or glycolysis) (130), engineered T cells (e.g., FX-engineered T cells) (113), and local delivery systems (e.g., IL-15 nanogel) (113) hold promise for achieving disease-specific immune regulation. In tumor immunotherapy, combining immune checkpoint inhibitors, vaccines, and metabolic modulators can enhance Trm cell function; in autoimmune diseases, targeting metabolic vulnerabilities (e.g., Fabp4/5) or chemokine receptors (e.g., CXCR6, CCR8) of Trm cells can reduce their pathological infiltration. By integrating single-cell multi-omics, spatial transcriptomics, and artificial intelligence predictive models, dynamic analysis of Trm cell behavior and intelligent design of personalized treatment strategies may be achieved in the future. This will ultimately propel immunotherapy toward greater precision and safety.
